# Testosterone Inhibits Lipid Accumulation in Porcine Preadipocytes by Regulating *ELOVL3*

**DOI:** 10.3390/ani14152143

**Published:** 2024-07-23

**Authors:** Fuyin Xie, Yubei Wang, Shuheng Chan, Meili Zheng, Mingming Xue, Xiaoyang Yang, Yabiao Luo, Meiying Fang

**Affiliations:** 1 Department of Animal Genetics and Breeding, National Engineering Laboratory for Animal Breeding, MOA Key Laboratory of Animal Genetics and Breeding, Beijing Key Laboratory for Animal Genetic Improvement, State Key Laboratory of Animal Biotech Breeding, Frontiers Science Center for Molecular Design Breeding, College of Animal Science and Technology, China Agricultural University, Beijing 100193, China; fuyin939@163.com (F.X.); 15380345581@163.com (S.C.); mingmxue@163.com (M.X.); yangxy95v1@163.com (X.Y.); 2 Sanya Research Institute, China Agricultural University, Sanya 572025, China; pq007007@hotmail.com; 3 Beijing General Station of Animal Husbandry, Beijing 100107, China; 15652827528@163.com

**Keywords:** pig, testosterone, *ELOVL3*, lipid accumulation, androgen receptor

## Abstract

**Simple Summary:**

Castration induces fat deposition in boars and decreases carcass quality, prompting extensive research on the regulatory mechanism of fat deposition in castrated boars from a genetic perspective. Consequently, this study aimed to conduct a series of molecular biological experiments to elucidate how testosterone inhibits lipid droplet accumulation in porcine preadipocytes through *ELOVL3*. The findings from this study offer crucial data supporting genetic analyses of porcine fat deposition due to castration, thereby significantly contributing to enhancing pork quality and improving pig production efficiency.

**Abstract:**

Castration is commonly used to reduce stink during boar production. In porcine adipose tissue, castration reduces androgen levels resulting in metabolic disorders and excessive fat deposition. However, the underlying detailed mechanism remains unclear. In this study, we constructed porcine preadipocyte models with and without androgen by adding testosterone exogenously. The fluorescence intensity of lipid droplet (LD) staining and the fatty acid synthetase (*FASN*) mRNA levels were lower in the testosterone-treated cells than in the untreated control cells. In contrast, the mRNA levels of adipose triglycerides lipase (*ATGL*) and androgen receptor (*AR*) were higher than in the testosterone-treated cells than in the control cells. Subsequently, transcriptomic sequencing of porcine preadipocytes incubated with and without testosterone showed that the mRNA expression levels of very long-chain fatty acid elongase 3 (*ELOVL3*), a key enzyme involved in fatty acids synthesis and metabolism, were high in control cells. The siRNA-mediated knockdown of *ELOVL3* reduced LD accumulation and the mRNA levels of *FASN* and increased the mRNA levels of *ATGL*. Next, we conducted dual-luciferase reporter assays using wild-type and mutant *ELOVL3* promoter reporters, which showed that the *ELOVL3* promoter contained an androgen response element (ARE); furthermore, its transcription was negatively regulated by *AR* overexpression. In conclusion, our study reveals that testosterone inhibits fat deposition in porcine preadipocytes by suppressing *ELOVL3* expression. Moreover, our study provides a theoretical basis for further studies on the mechanisms of fat deposition caused by castration.

## 1. Introduction

Castration is used in pig production to remove taint. However, it leads to fat deposition and excessive fat tissue impacts meat quality and reduces pork production efficiency, ultimately leading to profit losses. We previously showed that the weight, backfat thickness, and abdominal fat weight of castrated pigs were significantly higher than those of intact pigs [1]. Currently, two types of castration are used: immune and surgical. Both aim to reduce testosterone release [2]. Pig castration causes a dramatic decrease in testosterone expression, which slows muscle growth and increases fat accumulation. This phenomenon is observed not only in pigs but also in other species. Several studies have demonstrated a negative correlation between obesity and the levels of free testosterone, bioavailable testosterone (free and albumin-bound), and total testosterone (free, bioavailable, and bound to sex-hormone-binding globulin [SHBG]), which is maintained in all age groups [3,4,5,6,7]. These data indicate a strong relationship between testosterone and fat deposition. However, the molecular mechanism underlying the association between testosterone and fat deposition in pigs remains unclear.

ELOVL3 is a family member of the elongases of very long-chain fatty acids (ELOVLs), and it is mainly expressed in the liver and adipose tissue and catalyzes the production of saturated and unsaturated long-chain fatty acids, such as C16, C18, and C20 [8,9,10,11]. The roles of *ELOVL3* in the skin, adipose tissue, and the liver have been extensively studied [12,13,14,15,16]. *ELOVL3*-ablated mice showed impaired formation of triglycerides and LD in the skin and brown adipose tissue [15,16]. Activation of *ELOVL3* expression is associated with increased fatty acid (FA) oxidation, and, during cold exposure, the enzyme functions in brown fat cells to replenish the intracellular pool of FAs and maintain lipid homeostasis [16]. *ELOVL3* also directly regulates FA composition in subcutaneous white adipose tissue [17], and *ELOVL3* expression and fat deposition are positively correlated. However, the association between *ELOVL3* and testosterone remains unclear. Therefore, we aimed to explore the mechanism underlying obesity caused by testosterone deficiency and the relationship between *ELOVL3* expression and fat deposition.

In the present study, we treated porcine preadipocytes with and without testosterone as cellular models of intact and castrated pigs and conducted a transcriptomic analysis of porcine preadipocytes incubated with and without testosterone to evaluate the role of *ELOVL3* in the regulation of the castration-mediated fat deposition. Our findings provide a molecular basis for testosterone-mediated regulation of lipid metabolism.

## 2. Material and Methods

### 2.1. Cell Culture, Differentiation, and Testosterone Treatment

Porcine preadipocytes [18] were cultivated in growth medium (GM) consisting of DMEM/F12 supplemented with 10% fetal bovine serum (Gibco, New York, NY, USA) and 1% penicillin-streptomycin solution (Gibco) at 37 °C in a 5% CO_2_ humidified environment. We previously showed that 50 nM testosterone (Sigma, Ronkonkoma, NY, USA) significantly inhibited adipogenic differentiation of 3T3-L1 cells (Figure S1). Differentiation preadipocytes (at 100% confluence) was induced by incubating the cells in adipogenic medium (GM containing 0.25 mM 3-isobutyl-1-methylxanthine (Sigma), 1 µM dexamethasone (Sigma), and 5 mg/mL insulin (Sigma)) for 3 d with or without testosterone, followed by another 3 d incubating in fresh medium (GM containing 5 mg/mL insulin) with or without testosterone. In some experiments, cells were treated with 200 nM flutamide (Sigma) [19]. Every 3 d, the medium was replaced with fresh medium containing the same concentrations of testosterone or flutamide. 

### 2.2. Oil Red O Staining

Oil Red O was used to stain adipocytes as previously described [20]. The cells were fixed with 4% paraformaldehyde (Solarbio, Beijing, China) for 20 min and rinsed twice with PBS (Gibco). The cells were then stained with Oil Red O solution for 20 min. Finally, the stained cells were examined under a microscope.

### 2.3. BODIPY 493/503 Staining

Living cells were incubated with 4% paraformaldehyde for 20 min and then stained with BODIPY staining (Beyotime Biotechnology, Shanghai, China) solution for 15 min at 37 °C in the dark. Next, the cells were washed with PBS and incubated with DAPI (Beyotime Biotechnology) for 10 min. Finally, the cells were analyzed under a fluorescence microscope.

### 2.4. Quantitative RT-PCR

Total RNA was extracted from cells using TRIzol^®^ reagent (Invitrogen, Carlsbad, CA, USA) and was reverse transcribed to obtain cDNA. The obtained cDNA was used as a template for qRT-PCR along with Taq Pro Universal SYBR qPCR Master Mix (Vazyme Biotech Co., Nanjing, China) and specific primers in a qTOWER 2.1 thermocycler (Analytikjene, Jena, Germany). The housekeeping gene β-actin was used as a control for normalization [21]. Primers for qRT-PCR were designed using the NCBI website and are listed in Table S1.

### 2.5. Transcriptomic Analysis of Porcine Preadipocytes Incubated with and without Testosterone

Cells were separately harvested on 6 d in the testosterone and control groups (*n* = 3 per group). Total RNA was extracted with TRIzol^®^ reagent and then quantified and quality-checked using a Nanodrop. The mRNA library was built using 5 µg of RNA from six samples. The Illumina NovaSeq 6000 platform was used to build mRNA sequencing libraries. Sequenced data were quality-controlled, filtered, and mapped to the pig reference genome (Sus scrofa11.1) in HiSAT2. Gene expression was calculated in featureCounts and normalized to transcripts per million mapped reads (FPKM). The threshold for differentially expressed genes (DEGs) was set as adjusted *p* < 0.05 and |log2foldchange| > 1.

### 2.6. Small Interfering RNA Transfection

Three small interfering (si)RNAs targeting *ELOVL3* were designed and synthesized commercially (GenePharma, Suzhou, China); their sequences are shown in Table S2. Cells were transfected with the siRNAs using Lipofectamine 2000 reagent (Invitrogen, Carlsbad, CA, USA) according to the manufacturer’s instructions. Briefly, preadipocytes were plated in 12-well plates and allowed to grow to 90% confluence. Cells were incubated for 30 min in Opti-MEM and transfected with siRNA (120 nM) using Lipofectamine 2000 reagent. A scrambled siRNA, also designed by GenePharma, was used as a negative control.

### 2.7. Plasmid Constructs, and Cell Transfection

The full-length cDNA (2690 bp) of *AR* (GenBank: NM_214314) was amplified by overlapping PCR using cDNA extracted from porcine ovarian granulosa cells as a template and was cloned into the pcDNA3.1-EGFP vector (YRgene, Changsha, China). The primers used for PCR amplification are listed in Table S3. We also co-transfected 293 T cells [21] with 500 ng of five different 5′ deletion constructs (−2000 bp/+100 bp, −1460 bp/+100 bp, −920 bp/+100 bp, and −380 bp/+100 bp, and −158 bp/+100 bp) or the pGL3-Basic vector (Promega, Beijing, China) and pRL-TK (Promega, Beijing, China) plasmid using Lipofectamine 2000 reagent for 48 h. Similarly, 293 T cells were co-transfected with pGL3-*ELOVL3* wild-type or mutant plasmids and pcDNA3.1-*AR* or pcDNA3.1-EGFP vector for 48 h.

### 2.8. Cloning of the ELOVL3 Promoter Region and Bioinformatics Analysis

The porcine *ELOVL3* 5′-flanking sequence was amplified from porcine heart genomic DNA using 1.1 × S4 Fidelity PCR Mix (Genesand, Beijing, China). Primers used for PCR amplification are listed in Table S4 (−2000 to +100 bp; 2100 bp). The amplified product was cloned into the pGL3-Basic vector and sequenced (Sangon Biotech, Shanghai, China). Transcription factor binding sites in the *ELOVL3* promoter were predicted using PROMO (https://alggen.lsi.upc.es/cgi-bin/promo_v3/promo/promoinit.cgi?dirDB=TF_8.3 (accessed on 1 March 2023)) and the JARPAR database (https://jaspar.genereg.net/ (accessed on 1 March 2023)).

### 2.9. Deletion Analysis and Plasmid Construction

PCR primers were designed to hybridize at the −2000, −1460, −920, −380, and −158 bp positions to generate corresponding 5′ deletion derivatives using a common downstream primer at +100 bp (the 5′ deletion primers are listed in Table S4). The resulting amplicon was cloned into the pGL3-Basic vector using the KpnI/HindIII (NEB, Ipswich, MA, USA) sites. All plasmids were confirmed through DNA sequencing.

### 2.10. Site-Directed Mutagenesis

Overlap extension PCR was performed to generate site-directed mutants of the *ELOVL3* promoter. Primers were designed to replace the predicted transcription factor binding sites with complementary sequences of ARE (the primer sequences are listed in Table S4). PCR generated two DNA fragments containing the designated mutations in the overlapping regions. Subsequently, the two DNA fragments were pooled as PCR templates to generate a full-length DNA fragment. The resulting amplicons were ligated into the pGL3-Basic vector into the KpnI and NheI-H (NEB) sites to create mutant constructs. Each mutant construct was confirmed through DNA sequencing.

### 2.11. Dual-Luciferase Reporter Assay

After 48 h incubation post-transfection, 293 T cells were harvested and lysed to measure luciferase activity. Relative luciferase activity was calculated as the ratio of firefly luciferase to renilla luciferase activity as determined using the Dual-Luciferase Reporter Assay System (Vazyme Biotech Co.) and a microplate reader, according to the manufacturer’s instructions.

### 2.12. Statistical Analysis

All treatments were conducted with three biological replicates. The significance of differences was assessed using a *t*-test and one-way ANOVA in SPSS software (version 26.0; SPSS Inc., Chicago, IL, USA). Data are presented as means ± SEM. Results of the *t*-test are denoted by asterisks: * *p* < 0.05, ** *p* < 0.01, *** *p* < 0.001 compared to the control (on the bars) or between the indicated groups. Results of one-way ANOVA are indicated by letters. The absence of significant differences is indicated by identical letters (*p* > 0.05), while different lowercase letters denote significant differences (*p* < 0.05), and different capital letters signify highly significant differences (*p* < 0.01).

## 3. Result

### 3.1. Testosterone Treatment Inhibited Fat Accumulation and Altered the Expression of Lipid Metabolism-Related Genes

**Figure 1:**
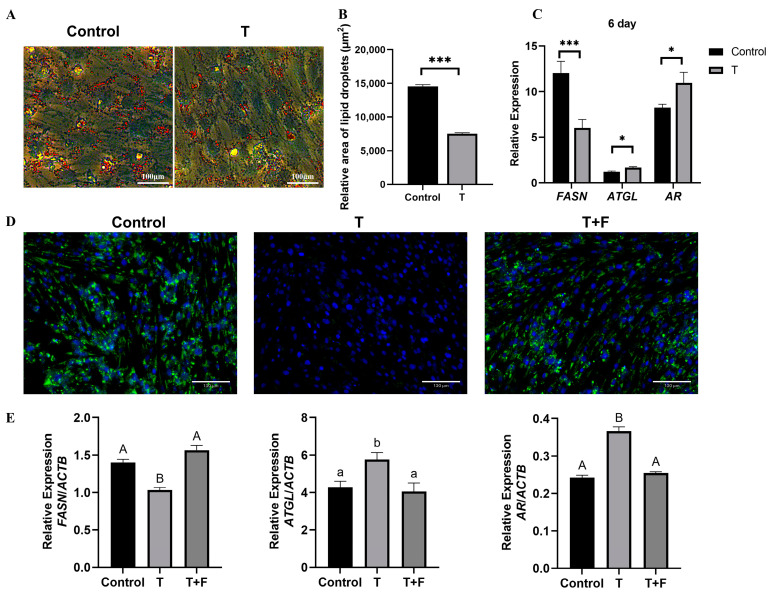
Testosterone treatment inhibited fat accumulation and altered the expression of lipid metabolism-related genes: (**A**) Oil Red O was used to stain porcine preadipocytes on the 6 d in the testosterone (T) and control groups (bar = 100 μm). (**B**) Quantification of LDs using Oil Red O analysis on the 6 d in the testosterone (T) and control groups. (**C**) The levels of *FASN*, *ATGL,* and *AR* mRNA in T group and control group on the 6 d of adipogenic differentiation of porcine preadipocytes were analyzed using quantitative RT-PCR. (**D**) LDs stained with BODIPY 493/503 in the T, testosterone + flutamide (T + F), and control groups (bar = 130 μm); (**E**) Quantitative RT-PCR analysis of the mRNA levels of *FASN*, *ATGL,* and *AR* on the 6 d in the T, T + F, and control groups. *n* = 3 per group. Statistical comparisons were performed with a *t*-test with one-way ANOVA. All data are expressed as means ± SEM. Results of the *t*-test are denoted by asterisks: * *p* < 0.05, *** *p* < 0.001 compared to the control (on the bars) or between the indicated groups. Results of one-way ANOVA are indicated by letters. The absence of significant differences is indicated by identical letters (*p* > 0.05), while different lowercase letters denote significant differences (*p* < 0.05), and different capital letters signify highly significant differences (*p* < 0.01).

Oil Red O staining showed that testosterone inhibited the LD accumulation during adipogenic differentiation of porcine preadipocytes (Figure 1A,B). After incubation of porcine preadipocytes with testosterone for 6 d, *FASN* mRNA expression was down-regulated, and *ATGL* and *AR* mRNA expression was up-regulated (Figure 1C). Flutamide, a selective AR antagonist, markedly attenuated the inhibitory effects of testosterone on the adipogenic differentiation of porcine preadipocytes (Figure 1D,E).

### 3.2. Transcriptomic Profiles of Porcine Preadipocytes Incubated with Testosterone

**Figure 2:**
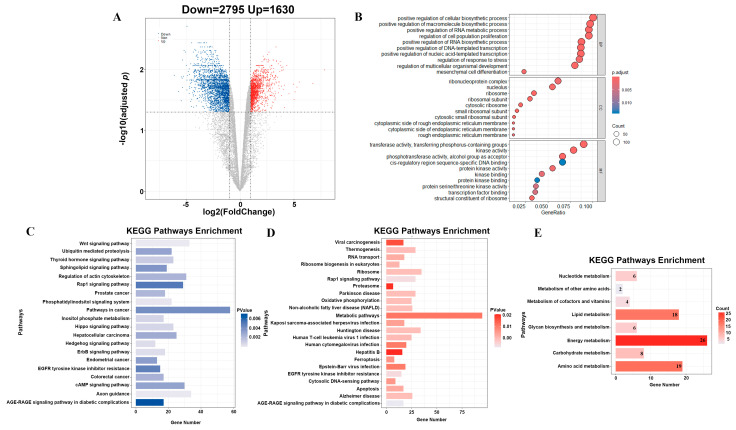
Transcriptomic profiles of porcine preadipocytes incubated with testosterone: (**A**) Volcano plot of the statistically significant DEGs (adjusted *p* < 0.05 and |log2FoldChange| > 1) between the testosterone-treated and control preadipocytes. (**B**) GO analysis of the DEGs. (**C**) KEGG analysis of the down-regulated DEGs; (**D**) KEGG analysis of the up-regulated DEGs; (**E**) KEGG analysis of metabolism-related DEGs. *n* = 3 per group.

To determine the molecular mechanism by which testosterone inhibits fat accumulation during adipogenic differentiation of porcine preadipocytes, we conducted transcriptomic sequencing of porcine preadipocytes treated with or without testosterone for 6 d. A comparison of the testosterone and control-treated cells identified 4425 DEGs (adjusted *p* < 0.05 and |log2FoldChange| > 1), including 1630 up-regulated genes and 2795 down-regulated genes (Figure 2A). To explore the functions of DEGs, we performed gene ontology (GO) and Kyoto Encyclopedia of Genes Genomes (KEGG) analyses. The results showed significant enrichment of GO terms related to cell proliferation, differentiation, and regulation of nucleic acid transcription, including RNA metabolism (positively regulated), RNA biosynthesis (positively regulated), and DNA template transcription (positively regulated), among other biological processes (Figure 2B). LDs are often observed adjacent to ER [22], and we observed a significant enrichment of GO entries related to cellular components associated with the ER as well (Figure 2B). The KEGG results showed that most DEGs were enriched in fat-related pathways, such as the wnt signal pathway, hedgehog signal pathway, and metabolic pathways (Figure 2C,D). Activation of wnt signaling and hedgehog signaling abolishes adipogenic differentiation by inhibiting the expression of adipogenic transcription factors [23]. Notably, the metabolic pathway possessed the most DEGs in the up-regulated differential gene KEGG enrichment analysis results, which was more than twice the number of genes in other enriched pathways. Further categorization of metabolic pathways showed that 18 up-regulated differential genes were significantly enriched in lipid metabolism pathways (Figure 2E). *ELOVL3*, a key gene involved in fatty acid synthesis and metabolism [16], was highly expressed in the control group (Tables S5 and S6). *AKR1C1* plays an important role in female obesity due to its unique progesterone metabolic characteristics [24]. Research on *PLA2G6* has predominantly focused on neurological diseases, and its relationship with androgen levels and fat deposition remains unexplored [25]. Consequently, we selected *ELOVL3* as a candidate gene for further investigation.

Taken together, these results suggest that testosterone plays a crucial role in adipogenesis through interactions with the DEGs and signaling pathways associated with adipogenesis and metabolism.

### 3.3. Knockdown of ELOVL3 Expression Inhibited Porcine Fat Accumulation

**Figure 3:**
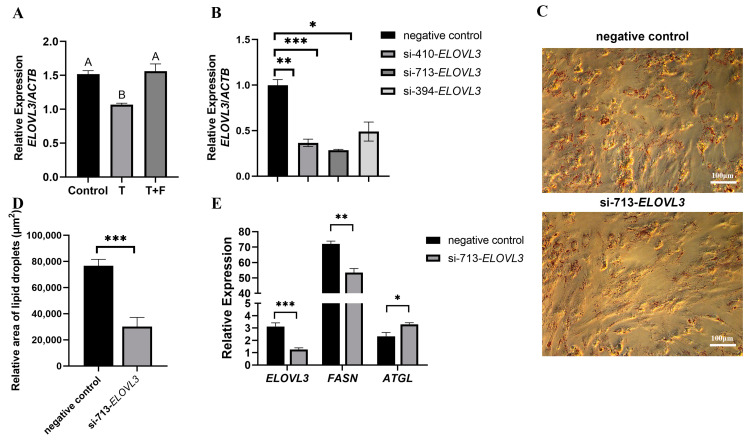
Knockdown of *ELOVL3* expression inhibited porcine fat accumulation: (**A**) *ELOVL3* mRNA levels in the different groups were tested with qRT-PCR. (**B**) Porcine preadipocytes were transfected with *ELOVL3* siRNAs (si-713-*ELOVL3*, si-394-*ELOVL3*, and si-410-*ELOVL3*) or a negative control siRNA, and *ELOVL3* expression interference efficiency was analyzed at 48 h after transfection. The relative expression of *ELOVL3* was normalized, and the relative values were expressed as the fold of induction relative to the negative control. (**C**) LD accumulation in preadipocytes transfected with either si-713-*ELOVL3* or a negative control siRNA was analyzed using Oil Red O staining at 6 d of induction (bar = 100 μm). (**D**) Quantification of LDs using Oil Red O analysis on the 6 d in the si-713-*ELOVL3* and negative control groups. (**E**) The mRNA levels of *ELOVL3*, *FASN*, and *ATGL* in the si-713-*ELOVL3* and negative control group were confirmed as measured using qRT-PCR. *n* = 3 per group. All data are expressed as means ± SEM. Results of the *t*-test are denoted by asterisks: * *p* < 0.05, ** *p* < 0.01, *** *p* < 0.001 compared to the control (on the bars) or between the indicated groups. Results of one-way ANOVA are indicated by letters. The absence of significant differences is indicated by identical letters (*p* > 0.05), while different lowercase letters denote significant differences (*p* < 0.05), and different capital letters signify highly significant differences (*p* < 0.01).

First, we verified the regulation of *ELOVL3* gene expression by testosterone. The *ELOVL3* gene expression was significantly down-regulated after being treated with testosterone, and the down-regulation of *ELOVL3* gene expression was rescued when treated with testosterone and flutamide (Figure 3A). Further, to explore the role of *ELOVL3* in the adipogenic differentiation of porcine preadipocytes, we designed three *ELOVL3* siRNAs (si-410-*ELOVL3*, si-713-*ELOVL3*, and si-394-*ELOVL3*) and determined their interference efficiency. The results showed that si-713-*ELOVL3* exhibited the strongest inhibitory effect (Figure 3B) and was chosen for all subsequent experiments. Oil Red O staining results showed that porcine preadipocytes transfected with si-713-*ELOVL3* accumulated significantly fewer LDs than the control group 6 days after the induction of differentiation (Figure 3C,D). Furthermore, qRT-PCR results showed that *ATGL* mRNA levels were significantly up-regulated in preadipocytes transfected with si-713-*ELOVL3*, whereas *FASN* mRNA levels were significantly down-regulated (Figure 3E). These results suggest that the knockdown of *ELOVL3* expression inhibited the adipogenic differentiation of porcine preadipocytes.

### 3.4. AR Targets the ELOVL3 Promoter and Inhibits Its Transcriptional Activity

**Figure 4:**
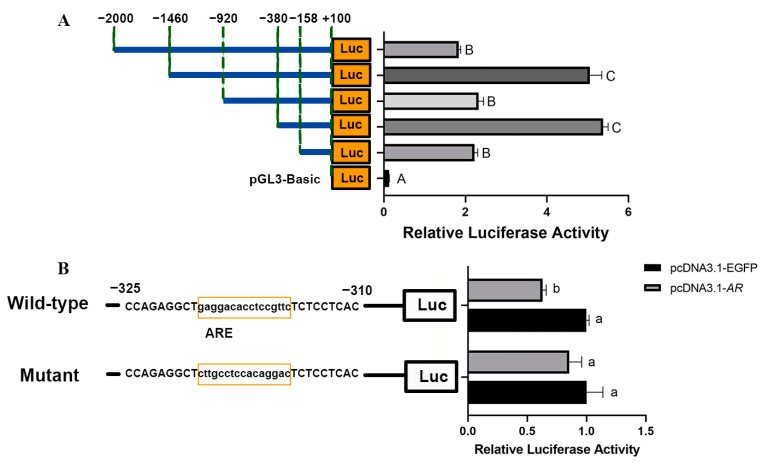
*AR* targets the *ELOVL3* promoter and inhibits its transcriptional activity: (**A**) Relative luciferase activity (Firefly: Renilla) at 48 h after transfection with different 5′ deletion *ELOVL3* promoter constructs (−2000 bp/+100 bp, −1460 bp/+100 bp, −920 bp/+100 bp, −380 bp/+100 bp, −158 bp/+100 bp). (**B**) Effects of *AR* overexpression on wild-type and mutant *ELOVL3* promoter activity. *n* = 3 per group. The luciferase activity was normalized, and the relative values were expressed as the fold of induction relative to the pcDNA3.1-EGFP vector activity. A one-way ANOVA test was used to assess the differences in luciferase activity. The absence of significant differences is denoted by the same letters (*p* > 0.05), while distinct lowercase letters indicate a significant difference (*p* < 0.05), and distinct capital letters signify a significant difference (*p* < 0.01).

It is well known that gene expression levels are directly related to promoter transcriptional activity. Transcriptome sequencing analysis after testosterone treatment showed differential genes significantly enriched in transcriptional regulation-related GO items. Therefore, we analyzed the promoter transcriptional activity of *ELOVL3*. We conducted five promoter fragments (−2000 bp/+100 bp, −1460 bp/+100 bp, −920 bp/+100 bp, −380 bp/+100 bp, −158 bp/+100 bp) and cloned them into the pGL3-Basic vector. Dual-luciferase reporter gene assay showed that deletion of the *ELOVL3* promoter (−2004 bp/−384 bp) significantly increased the promoter activity (*p* < 0.01), whereas the promoter activity decreased rapidly when the −384 bp/−158 bp region was deleted (*p* < 0.01; Figure 4A). This suggests that the −384 bp/−158 bp region is the essential promoter region of the *ELOVL3* gene. Testosterone normally exerts its biological effects by binding to the AR [26]. To determine whether *ELOVL3* is regulated by AR, the presence of ARE between −384 bp and −158 bp was predicted using PROMO and JARPAR databases. Moreover, 293 T cells were transiently co-transfected for 48 h with one of the two *ELOVL3* promoter luciferase structures (wild-type or mutant structure, predicted to be variable) and together with a porcine *AR* overexpression vector (pcDNA3.1-*AR*) or an empty vector (pcDNA3.1-EGFP). Dual-luciferase reporter gene assays showed that *AR* overexpression significantly repressed the activity of the *ELOVL3* promoter wild-type (*p* < 0.01) but not the mutant promoter (Figure 4B). This suggests that AR can regulate the transcriptional activity of the *ELOVL3* promoter region by directly targeting it and modulating its transcriptional activity.

## 4. Discussion

Testosterone is the main androgen in mammals and is secreted by Leydig cells [27]. In addition to its involvement in the development of male reproductive organs, testosterone also plays several important roles in adipose tissue [28]. Adipose tissue has been identified as an endocrine organ that secretes adipokines involved in metabolic and inflammatory pathways. Testosterone can affect adipocyte proliferation and differentiation, thereby affecting body fat composition, adipocyte function, and lipid metabolism [18]. Castration eliminates endogenous sources of testosterone production in the body. An analysis of castrated and intact pigs showed that castrated pigs exhibit lower serum testosterone levels and higher fat accumulation compared to intact pigs [29]. Numerous clinical and animal experiments have also confirmed that low testosterone levels can lead to massive fat deposition in the body [18]. To investigate the molecular mechanism underlying castration-induced lipid deposition in pigs, we incubated porcine preadipocytes with testosterone to simulate the androgen status of intact pigs. Here, we present data that demonstrate that testosterone inhibited the LD accumulation in porcine adipocytes and altered the expression of lipid metabolism-related genes, like *ATGL* and *FASN*. LDs are storage organelles at the center of lipid and energy balance [30]. The size and abundance of LDs determine their capacity for lipid storage. *ATGL* encodes an enzyme that promotes lipolysis in adipocytes, and *FASN* encodes a key enzyme involved in the de novo synthesis of fatty acids [31,32]. Lipid metabolism comprises two distinct processes: fatty acid β-oxidation and lipid synthesis [33]. FASN and ATGL are involved in these processes. Fat deposition results from an imbalance between lipid synthesis and lipolysis. These data indicate that testosterone regulates lipid metabolism to inhibit porcine subcutaneous fat deposition.

We conducted the transcriptomic sequencing of porcine preadipocytes incubated with and without testosterone for 6 d. Transcriptome data suggest that *ELOVL3* is a positive regulator of lipid accumulation in porcine preadipocytes. Rolf et al.’s data show that the elongase *ELOVL3* does affect the condensation reaction, which is the rate-limiting step for the elongation cycle of VLCFAs [15]. VLCFAs are important constituents of glycerophospholipids, sphingolipids, triglycerides, and sterol- and wax-esters [34]. Blocking *ELOVL3* expression with siRNA inhibited the expression of *FASN* and the LD formation and accumulation. Consistent with these findings, Zadravec et al. showed that *ELOVL3* knockout mice have reduced adiponectin levels, limited adipose tissue expansion, and resistance to diet-induced obesity [34].

Testosterone often exerts its effects through a classical pathway, in which testosterone binds to cytosolic AR, a ligand-activated transcription factor. Once activated, AR translocate into the nucleus to bind to AREs located in or near the promoter region of the target gene, thereby influencing its expression [35]. The expression of *AR* in pigs is tissue-specific, with high expression primarily observed in many endocrine glands, followed by adipose tissues, and relatively low expression in muscle and immunologic tissues [29]. AR plays an important role in the mechanisms of testosterone action in adipose tissue. Specific fat tissue AR knockdown (fARKO) mice developed metabolic dysregulation on a normal diet with early insulin resistance and hyperinsulinemia [36]. In our study, we observed that testosterone upregulated the expression of AR, consistent with findings reported by Liu in adipose tissue of control and castrated pigs [29]. Previous studies have demonstrated that the testosterone dose-dependent inhibition of lipogenic differentiation in 3T3-L1 cells could be partially blocked by flutamide, a selective AR inhibitor [19]. As a specific androgen antagonist, flutamide competitively inhibits androgen receptors and performs a direct blockage of androgenic effect [37]. In the present study, flutamide was found to ameliorate the inhibitory effect of testosterone on the LD accumulation in porcine preadipocytes, which suggested the involvement of AR signaling in androgen inhibition of porcine preadipocyte fat accumulation.

Transcriptome data suggest that testosterone deficiency contributes to the presence of *ELOVL3* in obesity. Our study involved an analysis of the activity within the promoter region of the *ELOVL3* gene, coupled with ARE prediction, revealing the presence of an ARE in the −384 to −158 region. Testosterone treatment led to an increase in the expression of *AR* while decreasing the expression of *ELOVL3*. The opposing effects observed on *AR* and *ELOVL3* expression suggest a potential negative regulatory role of *AR* on *ELOVL3.* Thus, we performed luciferase assays using a reporter vector, driven by the *ELOVL3* promoter, containing the wild-type AREs or mutated AREs. A dual-luciferase reporter assay showed that the overexpression of *AR* had no significant impact on the activity of the *ELOVL3* promoter when the predicted ARE was altered, indicating that the inhibitory effect of *AR* was abolished. These results indicated that *AR* inhibited activation of the *ELOVL3* promoter, and this down-regulation may require binding through the ARE in the *ELOVL3* promoter region. In conclusion, our findings support the hypothesis that testosterone may bind AR to suppress the expression level of *ELOVL3*.

Some limitations in our study, namely, the functional differences between porcine preadipocytes cultured in vitro and porcine preadipocytes grown in vivo, should be taken into account. Given the complexity of an organism’s environment, it is hard to fully mimic the characteristics of castration in vitro. In the current study, testosterone may affect the expression level of *FASN* and *ATGL* via AR by targeting *ELOVL3* and suppressing porcine fat deposition. The findings from this study enhance our comprehension of the mechanism behind increased fat deposition resulting from testosterone deficiency. Taken together, our in vitro porcine culture system provided a new possibility for establishing the in vitro model of fat accumulation in castrated boars; however, more evidence is needed to confirm this in vitro model.

## 5. Conclusions

**Figure 5:**
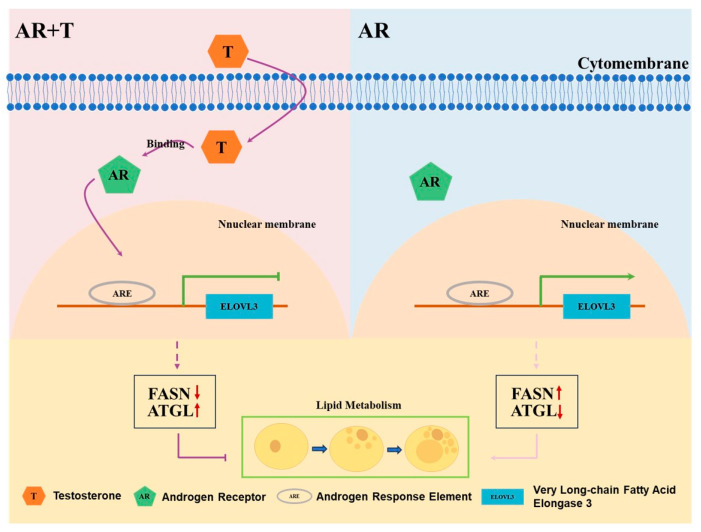
Testosterone inhibits lipid accumulation in porcine preadipocytes by regulating *ELOVL3*.

This study suggests that testosterone regulates *ELOVL3* to influence lipid metabolism and inhibit lipid droplet accumulation in porcine preadipocytes, potentially via the AR signaling pathway (Figure 5). However, further validation is needed to elucidate the precise molecular mechanism of *ELOVL3* in fat deposition in castrated boars. This research contributes to our understanding of how *ELOVL3* functions in fat deposition following testosterone depletion, providing both theoretical insights and empirical evidence for comprehending the molecular mechanisms underlying fat deposition due to castration in pigs.

## Data Availability

The data that support the findings of this study are available from the corresponding author upon reasonable request.

## Supplementary Materials

The following supporting information can be downloaded at: https://www.mdpi.com/article/10.3390/ani14152143/s1, Table S1: Characteristics of primers used in the qRT-PCR reaction; Table S2: *ELOVL3* small interfering RNA (siRNA) oligonucleotide sequences; Table S3: Primers used for constructing *AR* overexpression plasmid; Table S4: Primers used for cloning, deletion, and site-directed mutagenesis of *ELOVL3*; Table S5: Summary of 18 lipid metabolism-related genes identified through KEGG analysis; Table S6: KEGG analysis of *ELOVL3*; Figure S1: Effect of testosterone on adipogenic differentiation of 3T3-L1 cells.
