# Supplementary material for: Testosterone Inhibits Lipid Accumulation in Porcine Preadipocytes by Regulating ELOVL3
Source: Animals (Basel). 2024 Jul 23;14(15):2143. doi: 10.3390/ani14152143 (PMC11310965; doi:10.3390/ani14152143)
Supplement: Supplementary file 1 [file animals-14-02143-s001.zip › animals-3100407-supplementary - final version.pdf]

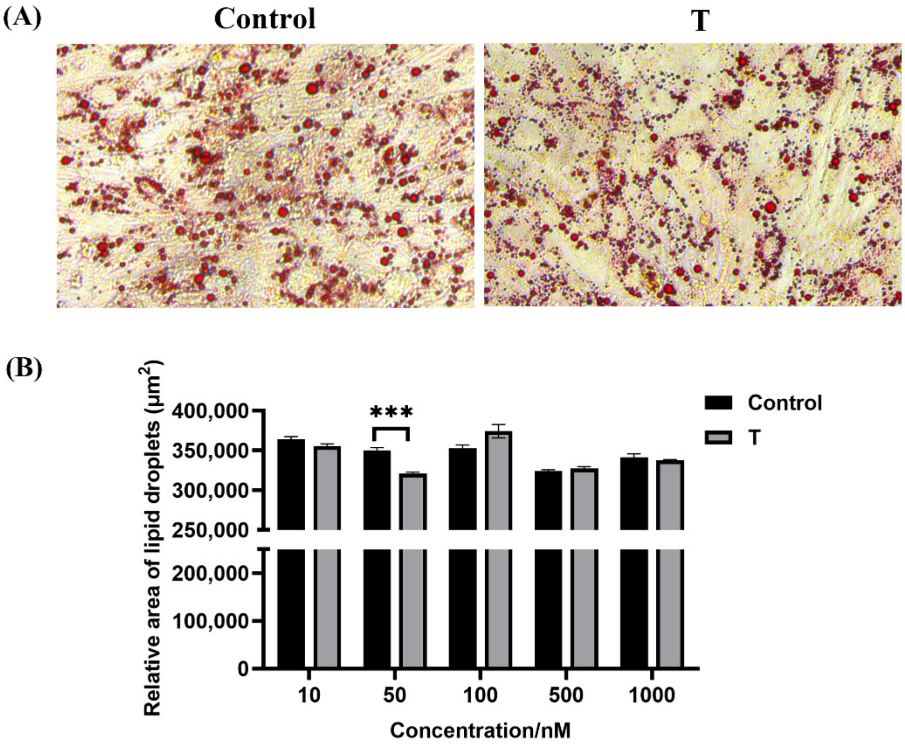

Supplementary Figure S1: Effect of testosterone on adipogenic differentiation of 3T3-L1 cells. (A) Oil Red O was used to stain 3T3-L1 cells on the 6 d in the testosterone (T) and control groups with different concentration (bar=100 μm); (B) Quantification of LDs by oil red o analysis on the 6 d in the testosterone (T) and control groups; *n*=3 per group.
